# CRISPR-based targeting of DNA methylation in *Arabidopsis thaliana* by a bacterial CG-specific DNA methyltransferase

**DOI:** 10.1073/pnas.2125016118

**Published:** 2021-05-31

**Authors:** Basudev Ghoshal, Colette L. Picard, Brandon Vong, Suhua Feng, Steven E. Jacobsen

**Affiliations:** ^a^Department of Molecular, Cell and Developmental Biology, University of California, Los Angeles, CA 90095;; ^b^Eli and Edythe Broad Center of Regenerative Medicine and Stem Cell Research, University of California, Los Angeles, CA 90095;; ^c^HHMI, University of California, Los Angeles, CA 90095

**Keywords:** DNA methylation, *Arabidopsis*, CRISPR-Cas9, SunTag

## Abstract

CRISPR-based targeted modification of epigenetic marks such as DNA cytosine methylation is an important strategy to regulate the expression of genes and their associated phenotypes. Although plants have DNA methylation in all sequence contexts (CG, CHG, CHH, where H = A, T, C), methylation in the symmetric CG context is particularly important for gene silencing and is very efficiently maintained through mitotic and meiotic cell divisions. Tools that can directly add CG methylation to specific loci are therefore highly desirable but are currently lacking in plants. Here we have developed two CRISPR-based CG-specific targeted DNA methylation systems for plants using a variant of the bacterial CG-specific DNA methyltransferase MQ1 with reduced activity but high specificity. We demonstrate that the methylation added by MQ1 is highly target specific and can be heritably maintained in the absence of the effector. These tools should be valuable both in crop engineering and in plant genetic research.

## Significance

Site-specific modification of epigenetic marks such as DNA methylation to regulate gene expression is a unique approach to enhance economically important crop traits. This approach allows for the maintenance of the introduced modifications in the absence of the initial transgene inducer in subsequent generations and relies largely on methylation of cytosines in the CG-specific sequence context. We have developed a targeted DNA methylation tool based on a bacterial methyltransferase and the CRISPR-Cas9 platform to directly methylate cytosines at CG sites in *Arabidopsis*. These tools expand the currently available CRISPR-based targeted DNA methylation tools and provide an approach for the establishment of heritable targeted DNA methylation in plants.

DNA methylation is a modification of the DNA base cytosine and is often associated with transcriptional repression. In plants, the RNA-directed DNA methylation (RdDM) pathway is required for establishment of de novo DNA methylation (1, 2), catalyzed by DOMAINS REARRANGED METHYLTRANSFERASE 2 (DRM2). Once established, DNA methylation is maintained via three main mechanisms, depending on genomic location and sequence context: CG methylation is maintained by DNA METHYLTRANSFERASE 1 (MET1) (1), non-CG methylation at small transposons and repetitive elements in open chromatin is maintained by RdDM, and non-CG methylation in dense heterochromatin is maintained by CHROMOMETHYLASE 2 (CMT2) and CHROMOMETHYLASE 3 (CMT3) (3, 4). Maintenance of methylation by RdDM in euchromatic regions is complex and depends on small interferring RNAs (siRNA) biogenesis, whereas methylation maintenance in pericentromeric regions by CMT2 and CMT3 depends on a feedback loop with heterochromatic histone modifications (1, 3). In contrast, symmetric CG methylation maintenance by MET1 during DNA replication depends on few additional factors and results in highly efficient copying of the methylation state from parent to daughter strand during cell division and meiosis (1, 4–6). Targeting of methylation directly to cytosines in the CG context should therefore be an effective strategy for establishing de novo DNA methylation that can be stably transmitted both mitotically and meiotically.

CRISPR-based systems have been used to target DNA methylation and trigger silencing of target loci in both plants and animals (7, 8). To date, the CRISPR-based tools developed in plants have used the catalytic domain of the RdDM methyltransferase DRM2, which adds DNA methylation in all sequence contexts but has a preference for the CHH context (8, 9). While these tools are able to cause gene silencing, they work at a relatively low efficiency, and the heritability of the targeted DNA methylation in the absence of the effector transgene is often incomplete (8). In a study utilizing an artificial zinc finger fused to RdDM components to target DNA methylation, Gallego-Bartolome et al. (10) demonstrated that the heritability of targeted DNA methylation was highly dependent on the establishment of high levels of CG methylation. To test the hypothesis that targeting methylation directly to cytosines in the CG context would increase silencing efficiency and heritability relative to the currently available tools, we sought to develop a CRISPR-based CG-specific targeted DNA methylation system for plants. A CG-specific bacterial methyltransferase, MQ1 (SssI), from the bacterium *Mollicutes spiroplasma*, fused to inactive Cas9 (dCAS9), was recently used to trigger de novo DNA methylation in the CG context at specific target loci in mammalian cell lines and mice (7). However, these lines also exhibited nonspecific gain of CG methylation throughout the genome (7). To reduce nonspecific methylation, Lei et al. (7) used a variant of MQ1 (MQ1^(Q147L)^) with reduced DNA binding affinity and activity. Here we show that this MQ1^(Q147L)^ variant can be used for accurate targeted de novo CG DNA methylation in *Arabidopsis*.

## Results and Discussion

### MQ1v Targets DNA Methylation to *FWA* Leading to *FWA* Silencing and an Early-Flowering Phenotype.

**Fig. 1.**
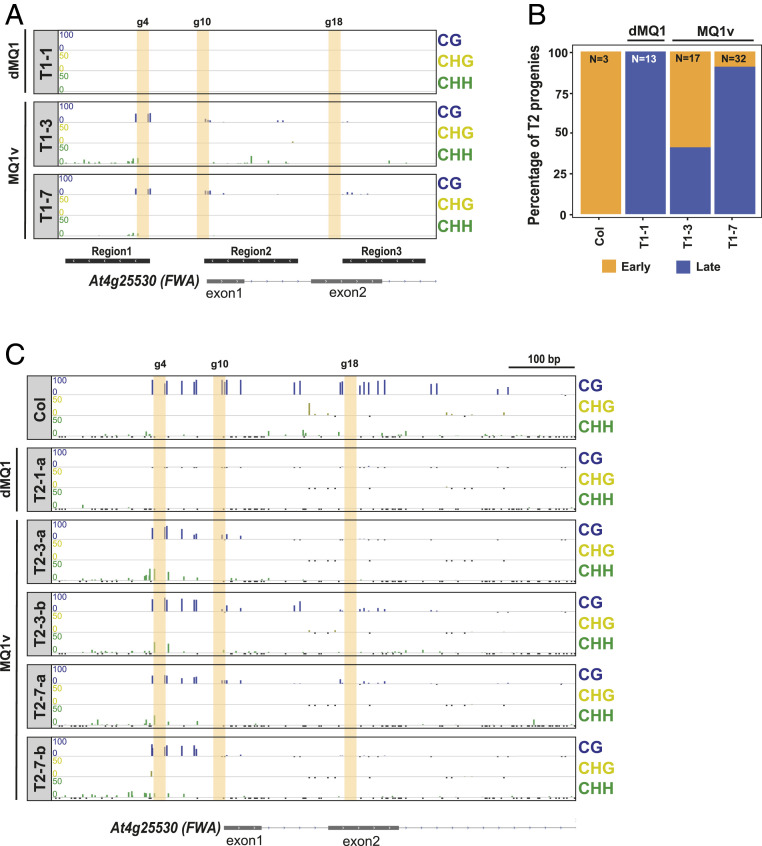
MQ1v targets DNA methylation to *FWA* and causes an early-flowering phenotype. (*A*) Bisulfite PCR sequencing of two T1 lines (T1-3, T1-7) transformed with MQ1v and a negative control (T1-1) transformed with dMQ1, over three regions of the *FWA* promoter region: region 1 (Chr4:13038143–13038272), region 2 (Chr4:13038356–13038499), and region 3 (Chr4:13038568–13038695). Each T1 plant is a result of an independent transgenic event. (*B*) Stacked bar plot showing the percentage of T2 early- and late-flowering plants in the progeny of two different T1 lines (T1-3 and T1-7), together with wild-type Col, and T2 progenies of a matched dMQ1 (T1-1) control. Percentage of early-flowering plants is denoted in the bar plots. The total numbers (N) of plants assayed for each line were 3, 13, 17, and 32. (*C*) DNA methylation profile at the *FWA* promoter region in early-flowering progeny of two T1 plants (T2-3-a, T2-3-b are progeny of T2-3; T2-7-a, T2-7-b are progeny of T2-7), along with Col and a matched dMQ1 (T2-1-a) plant as controls. Only cytosines with at least five overlapping reads are shown. Small, negative values (black) indicate cytosines with five or more overlapping reads but no DNA methylation. Vertical yellow bars indicate the locations of the guide 4, guide 10, and guide 18 binding sites.

We cloned dCAS9 fused to MQ1^(Q147L)^ (hereafter called MQ1v) into a plant binary vector (*SI Appendix*, Fig. S1*A*) and targeted MQ1v to the promoter of the *FLOWERING WAGENINGEN* (*FWA*) gene (11, 12). *FWA* is normally silent in the vegetative tissues of wild-type plants due to DNA methylation in its promoter region (13). However, in plants that have stably lost DNA methylation at the *FWA* promoter (*fwa* epimutants), *FWA* is expressed, causing a late-lowering phenotype. Thus, targeting the *FWA* promoter in *fwa* epimutants produces a simple phenotypic readout (flowering time) that is linked to the methylation status of the *FWA* promoter (8, 10, 14, 15). An MQ1v construct containing three previously characterized guide RNAs (gRNAs) (guide 4, guide 10, and guide 18) targeting three different regions of the *FWA* promoter (*SI Appendix*, Fig. S1*B*) (8) was transformed into an *fwa* epimutant (Col background) and T1 transformants were monitored for an early-flowering phenotype. As a negative control, we transformed *fwa* epimutant plants with a catalytically inactive mutant of MQ1 (dMQ1) (7) fused to dCAS9, along with the same three guide RNAs (*SI Appendix*, Fig. S1*A*). We did not observe any difference in flowering time between the first-generation transformed (T1) MQ1v and control dMQ1 plants, indicating that MQ1v could not trigger sufficient de novo DNA methylation to silence *FWA* in T1 plants. We examined DNA methylation levels in these T1 plants at the *FWA* promoter using a restriction-enzyme–based DNA methylation assay (McrBC–quantitative real-time PCR [McrBC-qPCR]). Out of eight MQ1v T1 plants tested, two (T1-3, T1-7) had gained DNA methylation in comparison to matched dMQ1 negative control plants (T1-1) (*SI Appendix*, Fig. S2*A*). To confirm this finding, we performed bisulfite PCR sequencing (BS-PCR) on both lines. We found that the two MQ1v T1 plants (T1-3, T1-7) had gained DNA methylation in both the CG and CHH contexts in the *FWA* promoter, compared to a control dMQ1 T1 plant (T1-1) (Fig. 1*A*). Thus, MQ1v was able to trigger de novo DNA methylation in these plants.

Several studies in plants have shown that minor gains of de novo DNA methylation can become amplified after multiple generations of inbreeding (16, 17). To test whether the two MQ1v T1 plants (T1-3, T1-7) with modest gains in DNA methylation exhibited further gains in the following generation, we analyzed the progeny (T2) of both T1 lines. We found several early-flowering T2 plants (Fig. 1*B* and *SI Appendix*, Fig. S2*B*), indicating successful *FWA* gene silencing. Using whole-genome bisulfite sequencing (WGBS), we examined DNA methylation at the *FWA* promoter in four of these early-flowering T2 plants (T2-3-a, T2-3-b, T2-7-a, T2-7-b) and their matched T2 dMQ1 control (T2-1-a) (see naming schema in *SI Appendix*, Fig. S1*C*). DNA methylation in these plants (Fig. 1*C*) was more pronounced than in their T1 parents (Fig. 1 *A* and *C*). To confirm that the gain of DNA methylation in these plants did not also occur genome-wide, we also examined DNA methylation levels by WGBS. We observed little change in genome-wide DNA methylation levels (*SI Appendix*, Fig. S3), indicating that MQ1v is able to specifically target DNA methylation at the *FWA* promoter with minimal off-target effects.

**Fig. 2.**
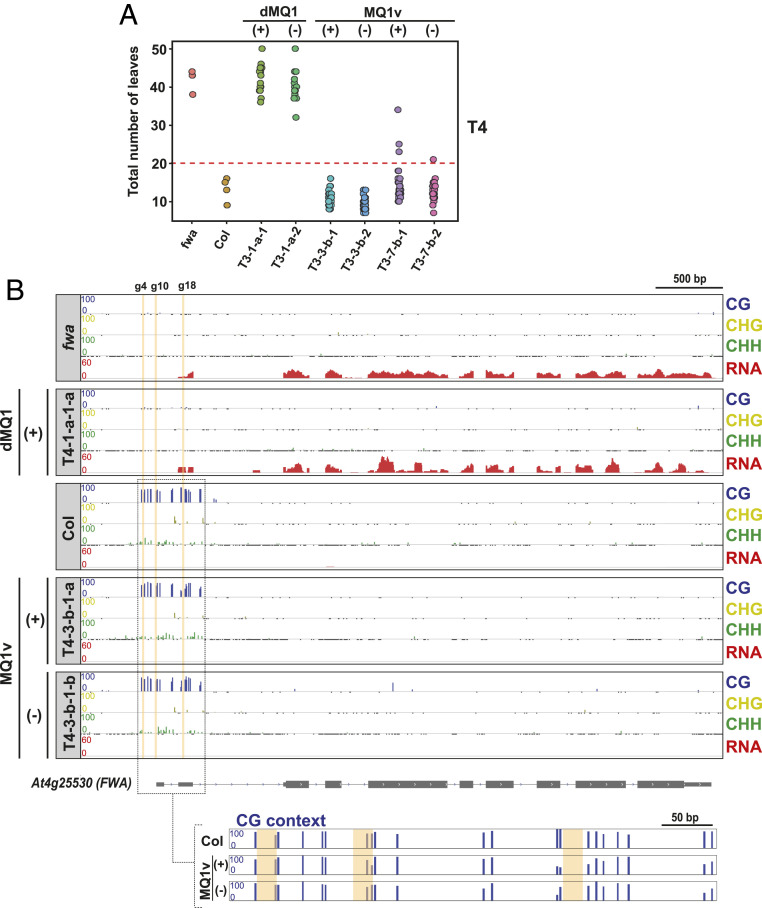
CRISPR-based targeted DNA methylation by MQ1v silences *FWA* expression and is heritable in the absence of the MQ1v effector. (*A*) Dot plot of leaf count at flowering time for four T4 MQ1v plants and two control dMQ1 plants. T3 parents are labeled at the bottom. (+), MQ1v transgene positive; (−), MQ1v transgene negative (null segregants). (*B*) DNA methylation profile at the *FWA* promoter region in T4 transgene positive (+) and T4 transgene negative (−) early-flowering plants, relative to Col wild-type control, the unmethylated *fwa* epiallele, and a late-flowering T4 dMQ1 control plant in the *fwa* background. *FWA* expression by RNA-seq is also shown (average of three replicates). (*Bottom*) Zoomed-in view shows strong DNA methylation at most of the CG sites in the targeted region. Vertical yellow bars indicate the locations of the guide 4, guide 10, and guide 18 binding sites.

Epigenetic marks such as DNA methylation can often be stably inherited in plants in the absence of the initial effector (8, 10, 15). We therefore examined whether MQ1v-targeted DNA methylation and the associated silencing of *FWA* were heritable in the absence of MQ1v. We first examined the progeny (T3) of the four early-flowering T2 plants (T2-3-a, T2-3-b, T2-7-a, T2-7-b) (*SI Appendix*, Fig. S4 *A* and *B*). The percentage of early flowering plants among the progeny of these T2 lines ranged from ∼14 to 100% (*SI Appendix*, Fig. S4*B*). We selected early-flowering T3 plants (*SI Appendix*, Fig. S4*A*) and used PCR-based genotyping to select two plants in which the MQ1v transgene was still present (T3-3-b-1, T3-7-b-1) and two plants in which the transgene had been segregated away (null segregants; T3-3-b-2, T3-7-b-2). These were propagated to the next generation (T4) and again monitored for the flowering-time phenotype (Fig. 2*A* and *SI Appendix*, Fig. S4*B*). Nearly all of the T4 plants were early flowering even after the MQ1v construct had been segregated away, indicating efficient maintenance of silencing of *FWA* expression in the absence of the original effector (Fig. 2*A* and *SI Appendix*, Fig. S4*B*). We also analyzed the DNA methylation profile at the *FWA* promoter in a T4 plant maintaining the transgene and a T4 null segregant [T4-3-b-1-a(+), T4-3-b-1-b(−), respectively] by WGBS. Methylation in these T4 plants was comparable to that in Col wild-type plants at the *FWA* promoter in both T4 plants tested (Fig. 2*B*). As expected, *FWA* expression was also strongly repressed in these two plants [T4-3-b-1-a(+), T4-3-b-1-b(−)] relative to the original *fwa* epimutant and dMQ1 control plants (Fig. 2*B* and *SI Appendix*, Fig. S4*C*). Thus, the DNA methylation and gene silencing targeted by MQ1v can be efficiently maintained in the absence of the transgene.

### MQ1v Can Cause Heritable DNA Methylation and Silencing in a *drm1 drm2* Mutant Background.

**Fig. 3.**
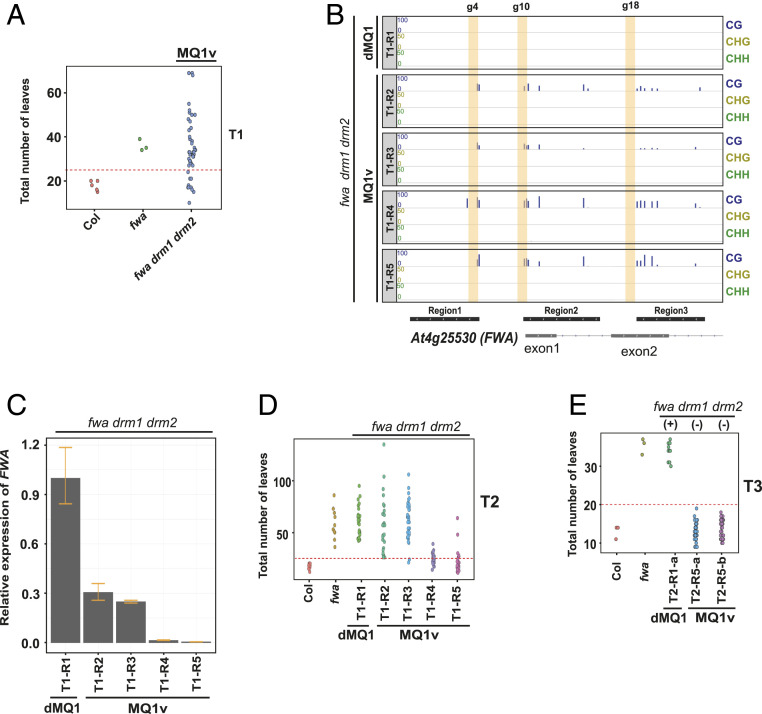
MQ1v can target DNA methylation in a *drm1 drm2* mutant background. (*A*) Dot plot of leaf count at flowering time of T1 plants transformed with dCAS9-MQ1v in *fwa drm1 drm2*, compared to *fwa* and Col controls. Each T1 plant is the result of an independent transgenic event. (*B*) Bisulfite PCR sequencing of four early-flowering T1 lines in *fwa drm1 drm2* (T1-R2, T1-R3, T1-R4, and T1-R5) over three regions of the *FWA* promoter: region 1 (Chr4:13038143–13038272), region 2 (Chr4:13038356–13038499), and region 3 (Chr4:13038568–13038695). A dMQ1 T1 plant was used as a negative control (T1-R1). Vertical yellow bars indicate the locations of the guide 4, guide 10, and guide 18 binding sites. (*C*) *FWA* expression analysis in four early-flowering MQ1v *fwa drm1 drm2* T1 plants relative to dMQ1 negative controls by reverse transcriptase–qPCR analysis. Error bars indicate SDs (*n* = 3 technical replicates). (*D* and *E*) Dot plot of leaf count at flowering time of T2 plants (*D*) and T3 plants (*E*) in the *fwa drm1 drm2* mutant background compared to *fwa* and Col controls. The T1 (*D*) or T2 (*E*) parent is listed at the bottom. In *E*, transgene positive (+) and transgene negative (−) lines are also indicated at the top.

Although MQ1 is a CG-specific DNA methyltransferase, MQ1v-transformed plants also gained non-CG methylation (Figs. 1*C* and 2*B*). This is likely due to the RdDM pathway, which is recruited to sites of existing DNA methylation and can add additional DNA methylation at both CG and non-CG sites, acting as a self-reinforcing feedback loop (1). Thus, initial establishment of CG methylation at the *FWA* promoter likely triggered additional de novo non-CG methylation via RdDM (Figs. 1*C* and 2*B*). However, silencing of *FWA* is known to be primarily dependent on CG methylation (18). To test whether MQ1v-mediated gain of CG methylation alone is sufficient to cause silencing of *FWA*, we tested MQ1v targeting in *fwa* epimutants in which *drm1* and *drm2* had been introgressed *(fwa drm1 drm2).* In this background, RdDM is abolished, and plants can no longer gain de novo non-CG methylation at *FWA* (10). We found that several T1 plants in the *fwa drm1 drm2* background were early flowering (Fig. 3*A*). We analyzed DNA methylation patterns at the *FWA* promoter by BS-PCR for four of these early-flowering plants (T1-R2, T1-R3, T1-R4, T1-R5) and a dMQ1 control plant (T1-R1). The *FWA* promoter was found to be methylated only in the CG context in these plants, as expected (Fig. 3*B*). Next, we examined *FWA* expression in the four early-flowering plants relative to the dMQ1 control plant and confirmed that *FWA* messenger RNA (mRNA) levels were suppressed in these plants (Fig. 3*C*). To determine whether loss of *DRM1* and *DRM2* affected the ability of *FWA* silencing to persist in the absence of the MQ1v transgene, we also monitored the T2 and T3 progeny plants of the four early-flowering T1 plants (T1-R2, T1-R3, T1-R4, T1-R5) for the early-flowering phenotype. Many T2 plants retained the early-flowering phenotype (Fig. 3*D*). Using PCR-based genotyping, we identified two T2 early-flowering plants that had segregated away the MQ1v transgene (T2-R5-a, T2-R5-b) and propagated them to the T3 generation. All of these T3 MQ1v plants retained the early-flowering phenotype (Fig. 3*E*), indicating that CG methylation alone was sufficient to maintain *FWA* silencing in an RdDM mutant background in the absence of the inducer.

It was somewhat paradoxical that *FWA* gene silencing was more efficient when MQ1v was transformed into the RdDM mutant background (*fwa drm1 drm2*) compared to the *fwa* background that contained a wild-type DNA methylation machinery, as indicated by the fact that early flowering plants could be seen in the T1 generation in *fwa drm1 drm2* plants (Fig. 3*A*), whereas MQ1v transformed into the *fwa* background caused *FWA* silencing only in the T2 generation (Fig. 1*B* and *SI Appendix*, Fig. S2*B*). This is likely due to increased accumulation of targeting components (MQ1v and/or guide RNAs) in the silencing-defective *drm1 drm2* mutant background. Transgenes are often targeted for repression by both transcriptional silencing machinery (TGS) and posttranscriptional silencing machinery (PTGS), namely the RdDM and RNA interference pathways (19, 20). For example, it was reported that PTGS mutants can increase both Cas9/gRNA expression and CRISPR-Cas9 editing efficiency (19). To test whether plants compromised in PTGS also show increased efficiency of methylation targeting by MQ1v, we transformed MQ1v into *fwa* plants that also carried *rdr6*, a PTGS pathway mutant (10). Similar to the *fwa drm1 drm2* background, we observed early-flowering phenotype in T1 plants (*SI Appendix*, Fig. S5*A*), associated with gain of DNA methylation at the *FWA* promoter (*SI Appendix*, Fig. S5*B*) as well as silencing of *FWA* expression (*SI Appendix*, Fig. S5*C*). T2 and T3 progenies of these lines also maintained the early-flowering phenotype in null segregants (*SI Appendix*, Fig. S5 *D* and *E*).

### Combining the SunTag System with MQ1^(Q147L)^ Robustly Targets DNA Methylation to *FWA* to Induce Early Flowering in T1 Plants.

**Fig. 4.**
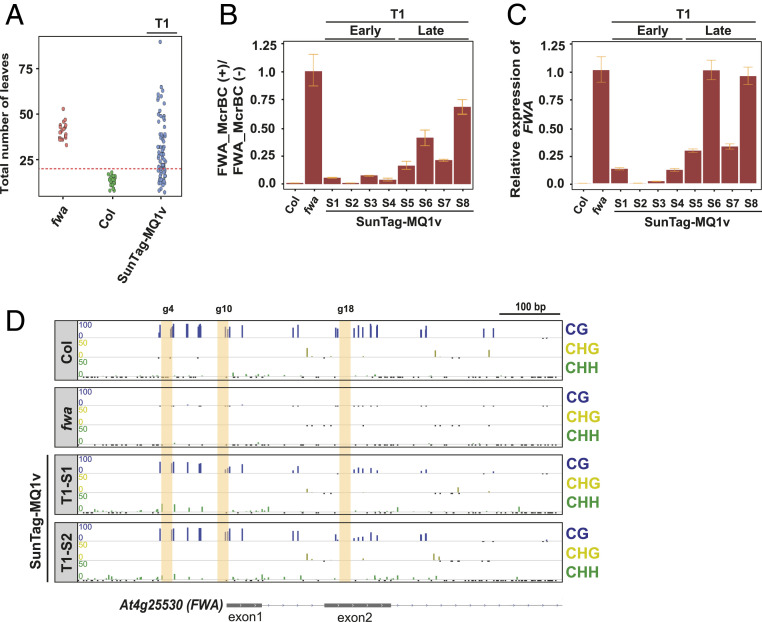
The CRISPR-based SunTag system is compatible with MQ1v and can target DNA methylation to *FWA* to induce early flowering. (*A*) Dot plot of leaf counts at flowering time of T1 plants transformed with SunTag-MQ1v. Each T1 plant is the result of an independent transgenic event. Leaf counts of Col and *fwa* plants are included as controls. (*B*) DNA methylation at *FWA* assayed by McrBC-qPCR in four T1 (T1-S1, T1-S2, T1-S3, T1-S4) early-flowering plants and four T1 (T1-S5, T1-S6, T1-S7, T1-S8) late-flowering plants by reverse transcriptase–qPCR analysis. Error bars indicate SDs (*n* = 3 technical replicates). (*C*) *FWA* expression analysis by qPCR for the same eight T1 plants analyzed in *B*. Error bars indicate SDs (*n* = 3 technical replicates). (*D*) DNA methylation profile at the *FWA* promoter region in two SunTag-MQ1v T1 plants (T1-S1, T1-S2) as determined by whole-genome bisulfite sequencing. Col and *fwa* are included in all the panels as controls. Vertical yellow bars indicate the locations of the guide 4, guide 10, and guide 18 binding sites.

We next examined ways to further increase the efficiency of MQ1-mediated targeted DNA methylation. The CRISPR-based SunTag system enables the recruitment of multiple effectors to the same target site, potentially increasing targeting efficiency compared to straight fusions with dCAS9 (21). We therefore tested whether using the SunTag system to target MQ1^(Q147L)^ to the *FWA* promoter led to more efficient gain of DNA methylation and *FWA* silencing. We cloned MQ1^(Q147L)^ into the SunTag construct (*SI Appendix*, Fig. S1*D*), hereafter called SunTag-MQ1v. We transformed *fwa* epimutants with SunTag-MQ1v and monitored the resulting T1 plants for the early-flowering phenotype. We observed many early-flowering plants in the T1 generation (Fig. 4*A*), indicating that SunTag-MQ1v is more efficient than the MQ1v straight fusion that showed only early-flowering plants in the T2 generation (Fig. 1*B* and *SI Appendix*, Fig. S2*B*). Four early-flowering T1 plants (T1-S1, T1-S2, T1-S3, T1-S4) and four late-flowering T1 plants (T1-S5, T1-S6, T1-S7, T1-S8) were selected for DNA methylation and *FWA* expression analysis. The four early-flowering T1 plants showed strong DNA methylation at the *FWA* promoter (Fig. 4*B*), along with a dramatic reduction in *FWA* mRNA levels relative to those in *fwa* (Fig. 4*C*). In contrast, the late-flowering plants showed little gain of DNA methylation and only partial *FWA* repression (Fig. 4 *B* and *C*). We also performed WGBS on two of the early-flowering T1 plants (T1-S1, T1-S2) and confirmed that these plants gained substantial DNA methylation at the *FWA* promoter (Fig. 4*D*). Both of these lines showed minimal changes in genome-wide DNA methylation levels (*SI Appendix*, Fig. S6). Together, these data demonstrate that MQ1^(Q147L)^ can be efficiently used with the SunTag system to target DNA methylation in *Arabidopsis* and is highly specific.

**Fig. 5.**
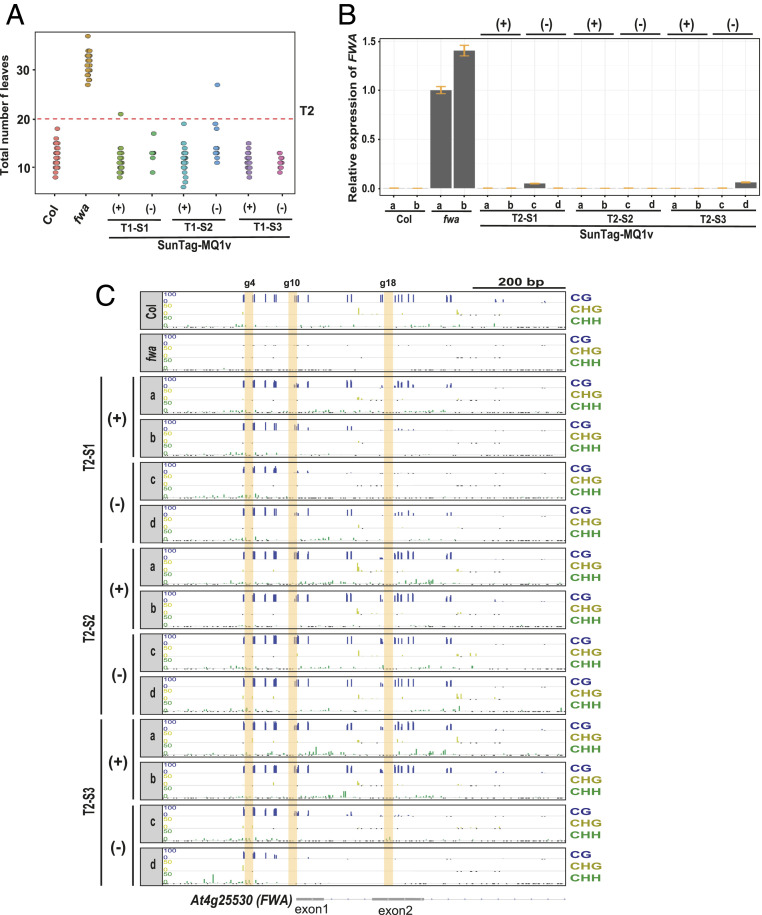
Heritability of targeted DNA methylation in SunTag-MQ1v T2 plants. (*A*) Dot plot of leaf counts at flowering time for T2 SunTag-MQ1v plants. T1 parents are labeled at the bottom. *fwa* and Col plants are included as controls. (*B*) *FWA* expression in *fwa*, Col, and early-flowering T2 SunTag-MQ1v plants both with (+) and without (−) the MQ1v transgene. (*C*) DNA methylation profile at the *FWA* promoter region in T2 transgene positive (+) and transgene negative (−) early-flowering plants, relative to a Col wild-type control and the unmethylated *fwa* epiallele. Vertical yellow bars indicate the locations of the guide 4, guide 10, and guide 18 binding sites.

Next, we examined whether the targeted methylation at *FWA* by SunTag-MQ1v was heritable in the absence of the (SunTag-MQ1v) effector transgene. We selected three T1 lines (T1-S1, T1-S2, T1-S3) and analyzed the progeny (T2 plants) of these plants for the early-flowering phenotype (Fig. 5*A*). Between 98 and 100% of the T2 plants from each line flowered early (*SI Appendix*, Fig. S7*A*). We selected two progeny plants for each line that still contained the transgene and two plants that had segregated away the transgene and then analyzed *FWA* expression and DNA methylation (Fig. 5 *A*–*C*). In accordance with the early-flowering phenotype, we observed repression of *FWA* expression and high levels of DNA methylation at the *FWA* promoter in these lines compared to the unmethylated *fwa* epimutants (Fig. 5 *B* and *C*). We also examined genome-wide DNA methylation levels in the T2 SunTag-MQ1v lines (*SI Appendix*, Fig. S8). All 12 T2 plants tested showed no or little change in genome-wide DNA methylation level in comparison to control *fwa* epimutants (*SI Appendix*, Fig. S8), indicating that SunTag-MQ1v is highly specific. To further test whether the early-flowering phenotype was maintained in the T3 generation, we examined the progeny of these 12 T2 plants. Nearly all progeny of these 12 plants flowered early (*SI Appendix*, Fig. S7*B*), indicating that DNA methylation and *FWA* silencing are efficiently maintained in the T3 generation even if the transgene was segregated away in an earlier generation. Together these results show that SunTag-MQ1v–targeted DNA methylation at *FWA* was both specific to the *FWA* locus and efficiently inherited in the absence of the effector transgene.

Previous studies have reported that DNA methylation can be amplified in subsequent generations (16, 17); in line with this, we observed that MQ1v-triggered and SunTag-MQ1v–triggered DNA methylation of *FWA* and early flowering were both more pronounced in later generations than in the T1 plants (Figs. 1, 2, 4, and 5). We also found that early-flowering SunTag-MQ1v T2 plants were common among the progeny of late-flowering T1s: Among the T2 progeny of seven SunTag-MQ1v T1 late-flowering plants, all lines tested gave rise to some early-flowering T3 plants, although the percentage varied widely (4 to 98%) (*SI Appendix*, Fig. S7*C*). This result further suggests that SunTag-MQ1v targeted methylation tends to amplify over successive generations.

### Cas9-MQ1v and SunTag-MQ1v Target a Similar Footprint of Methylation to That of *FWA*.

The SunTag system leads to the recruitment of multiple effector MQ1v proteins attached to an epitope tail while MQ1v recruits only a single MQ1 protein, which could potentially cause the “footprint” or region affected by SunTag to be larger than that of MQ1v. To compare the DNA methylation footprints, we selected lines that exhibited strong DNA methylation at the *FWA* promoter in progenies of MQ1v and SunTag-MQ1v plants. We observed almost identical DNA methylation footprints at *FWA* with both tools, with nearly all CG sites in the region showing methylation (*SI Appendix*, Fig. S9). This is likely due to a combination of factors. Notably, we found two regions flanking the target site at *FWA* that fully lack CG sites (*SI Appendix*, Fig. S9), which likely limit the range of both tools. Additionally, we observed CHH methylation over the target locus (Figs. 2*B* and 5*C*), indicating that the endogenous DNA methylation machinery, likely RdDM, is also being recruited to the target locus. Initial CG methylation seeded by MQ1v likely helps recruit these pathways, at which point local chromatin context and interactions with other factors would determine the full extent of targeted methylation. Thus, factors such as the genomic context and interactions with other pathways can influence the size of the region affected by these tools.

## Conclusion

We have developed two CRISPR-based systems, MQ1v and SunTag-MQ1v, that can directly install CG methylation at a specific locus. Our data suggest SunTag-MQ1v is more potent than MQ1v, and the differential strength of these two tools may provide the flexibility to induce different levels of de novo DNA methylation at different loci. These CRISPR-based tools are distinct from previously developed tools because they specifically target cytosines in a CG context, which likely improves heritability of the added DNA methylation at *FWA* due to the robustness of CG-methylation maintenance pathways. The CRISPR-based MQ1v systems described here were also highly specific, likely owing to the Q147L mutation that reduces the catalytic activity of the enzyme (7). Indeed, in a separate study, we found that an artificial zinc finger fused to the wild-type SssI enzyme caused broad off-target methylation throughout the genome (22).

Several factors may influence targeted DNA methylation. For example, we observed that the CG methylation added by MQ1v at *FWA* can eventually establish non-CG methylation, indicating the recruitment of other DNA methylation pathways. In plants, these pathways, including RdDM, are self-reinforcing (1) and likely help strengthen *FWA* silencing. However, when we transformed MQ1v into a null RdDM mutant background (*fwa drm1 drm2*), we found that methylation at CG sites alone was able to silence *FWA*, as previously shown (12). In fact, silencing was more efficient in the *drm1 drm2* background. This result was likely due to increased transgene expression rather than a direct consequence of loss of non-CG methylation at *FWA*, since a similar effect was observed in a background deficient in PTGS (*rdr6*). These results suggest that efficient transgene expression remains an important limiting factor in CRISPR-based epigenetic editing efforts, as has also been shown for CRISPR-based genome editing (19). This also demonstrates that for some loci, CG methylation is sufficient to silence the expression of a gene. For these loci, the tools described here are likely to be more efficient than the previously developed DRM2-based SunTag system (8). Overall, epigenetic targeting tools such as these may be useful in altering gene expression both for research applications and for crop improvement efforts.

## Materials and Methods

### Plant Materials and Growth Conditions.

*Arabidopsis thaliana* [ecotype Columbia-0 (col)] plants were grown under long-day conditions (16 h light/8 h dark) at 23 °C in controlled growth chambers. T1 transgenic plants were selected on 1/2 Murashige and Skoog (MS) medium + 35 μg/mL Hygromycin B (Invitrogen). Flowering time was measured by counting the total number of rosette and cauline leaves at the time of flowering. Red dashed lines in each dot plot of flowering time indicate the cutoff for considering a plant as early flowering, based on the flowering time of Columbia-0 plants that were grown side by side with the tested plants for each individual experiment (usually 20 or 25 leaves).

### Cloning dCAS9-MQ1v and SunTag-MQ1v Construct into Plant Binary Vector.

The MQ1v binary vector was generated by amplifying the dCAS9-MQ1^(Q147L)^ region from the pcDNA3.1-dCas9_MQ1(Q147L)EGFP construct (7). The amplified dCAS9-MQ1-147L was inserted between sites HpaI and AscI in the plant binary vector pEG302 that already contained UBQ10 promoter upstream of HpaI and OCS terminator downstream of AscI by using Infusion (Clontech). Guide RNAs were cloned in the KpnI site of the pEG302 vector by using Infusion (Clontech) (8). To clone MQ1v into the SunTag system, MQ1(Q147L) was amplified from the pcDNA3.1-dCas9_MQ1(Q147L)EGFP construct and was inserted into the BsiwI site of the pEG302-SunTag vector that was previously used in another study (8) and contained the three guide RNAs and the SunTag system by using Infusion (Clontech) following the manufacturer’s protocol.

### qPCR.

qPCR was performed as previously described (23). Rosette leaves were used for total RNA extraction by using a Direct-zol RNA Miniprep kit (Zymo) and using in-column DNase digestion. SuperScript III First-Strand Synthesis SuperMix (Invitrogen) was used for cDNA synthesis for reverse transcriptase qPCR. Oligos JP7911 (5′-tta​gat​cca​aag​gag​tat​caa​ag-3′) and JP7912 (5′- ctt​tgg​tac​cag​cgg​aga-3′) were used to detect *FWA* transcripts. The housekeeping gene *ISOPENTENYL PYROPHOSPHATE:DIMETHYLALLYL PYROPHOSPHATE ISOMERASE 2* (*IPP2*) was used to normalize the ct values. IPP2 transcripts were detected using oligos JP11859 (5′-gta​tga​gtt​gct​tct​cca​gca​aag-3′) and JP11860 (5′-gag​gat​ggc​tgc​aac​aag​tgt-3′). The delta ct values were calculated relative to the control plants.

### McrBC-qPCR.

McrBC-qPCR was performed as previously described (23). Briefly, the cetyl trimethylammonium chloride (CTAB) method was used to extract genomic DNA. McrBC restriction digestion was performed on 500-ng to 1-μg amounts of DNA for 4 h at 37 °C, followed by 20 min at 65 °C. As a negative control, equal amounts of DNA were incubated in buffer without the enzyme for 4 h at 37 °C. Oligos JP15049 (5′-*ttg​ggt​tta​gtg​ttt​act​tg*-3′) and JP15050 (5′-*gaa​tgt​tga​atg​gga​taa​ggt​a*-3′) were used for qPCR of the *FWA* promoter. The ratio between the digested and the undigested DNA in the samples was calculated using the qPCR data and was expressed relative to the control lines.

### WGBS Analysis.

The CTAB-based method was used to extract DNA. A total of 75 to 150 ng of DNA was used to prepare WGBS libraries by using the Ovation Ultralow Methyl-seq kit (NuGEN). Bisulfite treatment was performed by using the Qiagen EpiTect bisulfite kit per manufacturer’s instructions. Libraries were sequenced on Illumina HiSeq 4000 and NovaSeq 6000. Raw sequencing reads were aligned to the Arabidopsis genome (TAIR10) using Bismark (24), which was also used to generate per-position DNA methylation tracks. Reads with three or more consecutive methylated CHH sites were discarded since they are likely to be unconverted reads, as described previously (25). Browser images were obtained from Integrative Genomics Viewer (IGV) (26). Cytosines with a five or more read coverage and zero methylation were represented by a negative black bar, while cytosines with a less than five read coverage are not shown (no bar).

### Bisulfite PCR Sequencing.

Bisulfite PCR sequencing was performed as previously described (10, 23). Genomic DNA was extracted using the CTAB-based method followed by sodium bisulfite treatment of the DNA by the Qiagen EpiTect bisulfite kit per manufacturer’s instructions. Libraries were generated from purified PCR products amplified from the bisulfite-treated DNA by using previously described primers (10), using an Ovation Ultralow V2 kit (NuGEN) or a Kapa Kit (Roche) in combination with TrueSeq LT barcodes or IDT UD barcodes (Illumina). Illumina HiSeq 2500 was used to sequence the libraries. Bismark was used to map and process the bisulfite PCR reads (24). As with the WGBS analysis, reads with three or more consecutive methylated CHH sites were discarded since they are likely to be unconverted reads.

### Total RNA Sequencing.

Total RNA extraction was performed by using the Direct-zol RNA Miniprep kit (Zymo), using in-column DNase digestion. A total of 1 μg of RNA was used to prepare libraries by using the Illumina TruSeq Stranded Total RNA kit with Ribo-Zero Plant per manufacturer’s protocol and they were run on an Illumina HiSeq 4000 sequencer, with a single-end 50-bp protocol. Reads were first aligned to TAIR10 using the Spliced Transcripts Alignment to a Reference (STAR) package (27). Coverage tracks were generated using bamCoverage [deepTools, options–binSize 1–normalizeUsing CPM (28)], and replicates were then pooled using bigWigMerge from the University of California Santa Cruz (UCSC) toolkit (29). The number of reads mapping to each gene was also calculated by htseq-count (30) using default parameters. Differential expression analysis was performed using the DESeq2 software package (31). Predicted log2(fold change) values and SEs were extracted from the DESeq2 output for *FWA* (*SI Appendix*, Fig. S4*C*). However, due to low counts and high variability caused by failure of the depletion of the rRNA, further analysis of the DE results was not attempted.

## Supplementary Material

Supplementary File

## Data Availability

Next-generation sequencing raw data have been deposited in Gene Expression Omnibus (GEO) database, http://www.ncbi.nlm.nih.gov/geo (accession no. GSE149840). All study data are included in this article and/or *SI Appendix*.
