# Supplementary material for: CRISPR-based targeting of DNA methylation in Arabidopsis thaliana by a bacterial CG-specific DNA methyltransferase
Source: Proc Natl Acad Sci U S A. 2021 May 31;118(23):e2125016118. doi: 10.1073/pnas.2125016118 (PMC8201958; doi:10.1073/pnas.2125016118)
Supplement: Supplementary File [file pnas.2125016118.sapp.pdf]

Supplementary Information for

CRISPR-based targeting of DNA methylation in *Arabidopsis thaliana* by a bacterial CG-specific DNA methyltransferase.

Basudev Ghoshal[a,#], Colette L. Picard[a,#], Brandon Vong[a], Suhua Feng[a,b], Steven E. Jacobsen[a,b,c,1]

[#]contributed equally

[1]To whom correspondence should be addressed. E-mail: jacobsen@ucla.edu

[a]Department of Molecular, Cell and Developmental Biology, University of California, Los Angeles, CA 90095

[b]Eli and Edythe Broad Center of Regenerative Medicine and Stem Cell Research, University of California, Los Angeles, CA 90095

[c]Howard Hughes Medical Institute, University of California, Los Angeles, CA 90095

This PDF file includes:

Figures S1 to S9

Supplementary figures:

Figure S1.

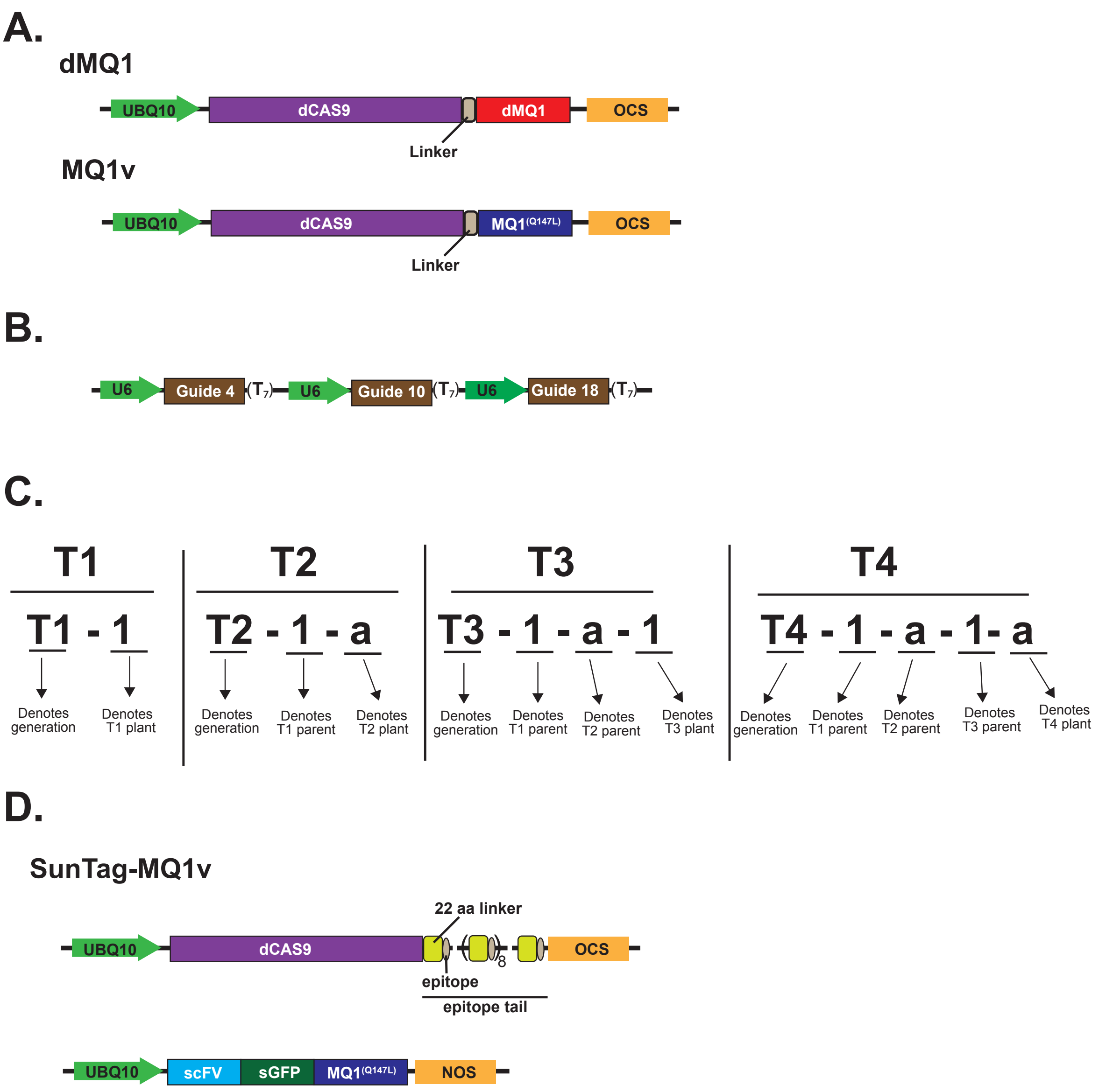

Figure S1. CRISPR-based methylation systems used with MQ1$^{(Q147L)}$ in this study to target DNA methylation A) Schematic representation of the direct fusion of catalytically inactive Cas9 (dCAS9) fused to MQ1$^{(Q147L)}$ (MQ1v) and a catalytically inactive mutant of MQ1 (dMQ1). Both fusions were expressed from a constitutive UBQ10 promoter with the OCS terminator. **B)** Schematic of guide RNA construct used to target constructs to *FWA*, consisting of three single guide RNAs (guide 4, guide 10, guide 18),

each with a PolII (U6) promoter and a stretch of poly (T)'s as a terminator. Guide 4 binds upstream of the transcription start site (TSS), guide 10 binds to the TSS, and guide 18 binds to the 5'UTR downstream of TSS (8). **C)** Naming schema for plants in T1, T2, T3, and T4 generations. **D)** Schematic representation of the SunTag system with MQ1$^{(Q147L)}$ (SunTag-MQ1v). In the SunTag system, the dCAS9 is fused to an epitope tail that contains ten peptide repeats. Each epitope is linked by a twenty-two amino acid linker (22aa). The MQ1$^{(Q147L)}$ effector is expressed as a fusion with the single-chain variable fragment (scFV) that can bind to the epitope.

Figure S2.

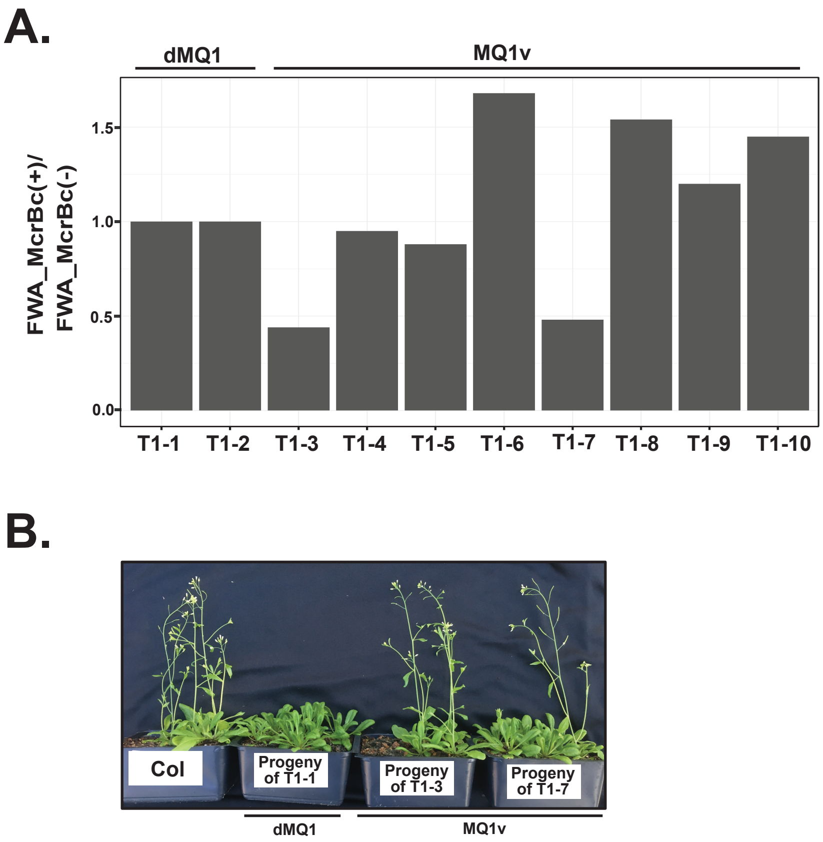

Figure S2. Targeted DNA methylation by MQ1v causes DNA methylation changes in T1 plants and early flowering in T2 plants. A) DNA methylation levels at *FWA* assayed by McrBC-qPCR for eight MQ1v plants in the T1 generation and two dMQ1 control plants. Each T1 plant is the result of an independent transgenic event. **B)** Examples of early-flowering T2 progeny of two T1 (T1-3, T1-7) plants, with Col wild type and T2 progeny of T1-1 (a dMQ1 control) as controls for early- and late-flowering phenotypes, respectively.

Figure S3.

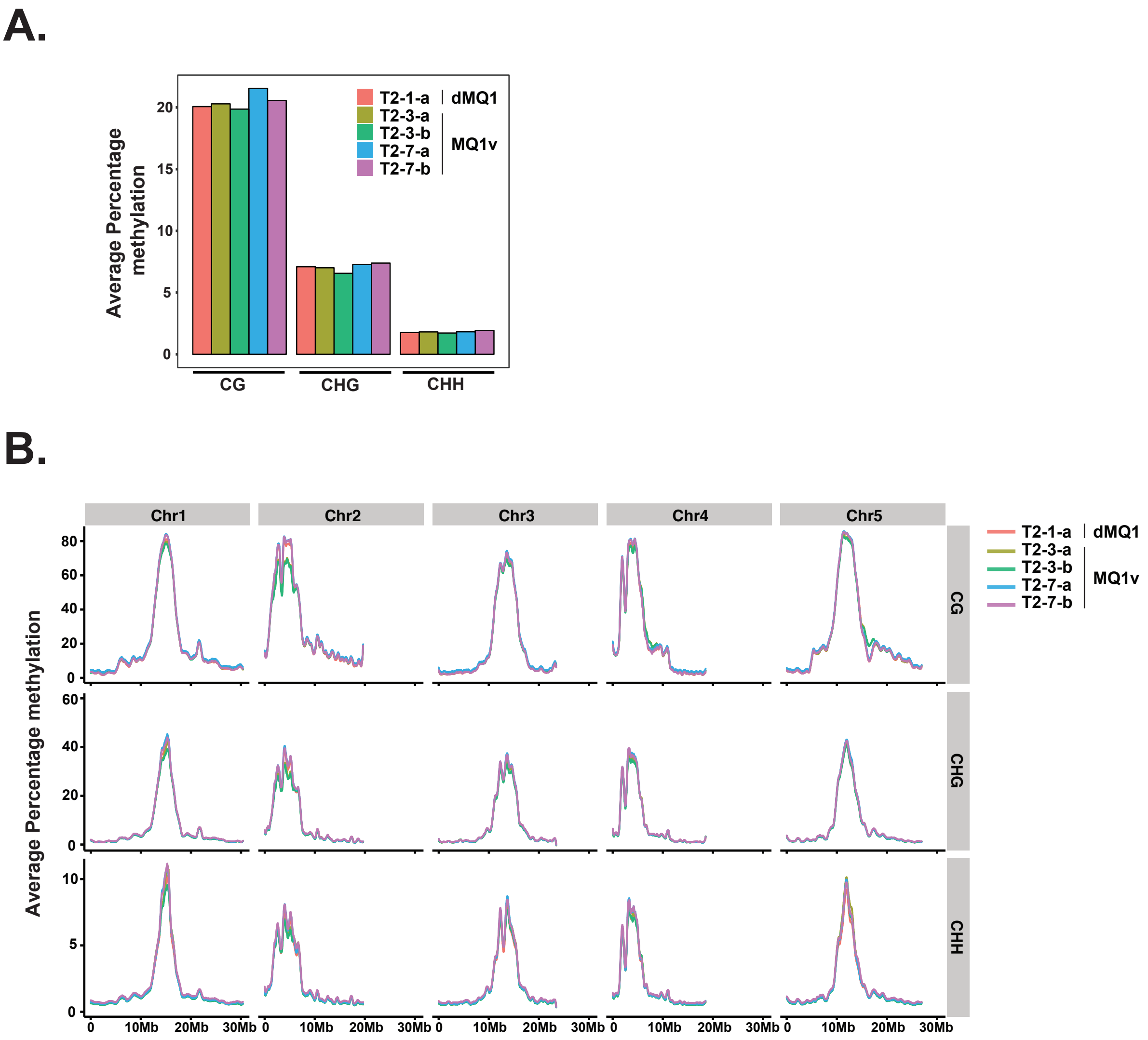

Figure S3. Genome-wide DNA methylation patterns in MQ1v transformed T2 plants relative to a dMQ1 control. Comparison of whole genome methylation patterns in four T2 early-flowering transgenic plants (T2-3-a, T2-3-b, T2-7-a, T2-7-b) compared to a dMQ1 matched control (T2-1-a). **A)** Bar graphs showing whole genome methylation percentages. **B)** Average methylation levels over each chromosome, obtained via locally weighted regression of average methylation levels over 50 kb bins (see methods).

Figure S4.

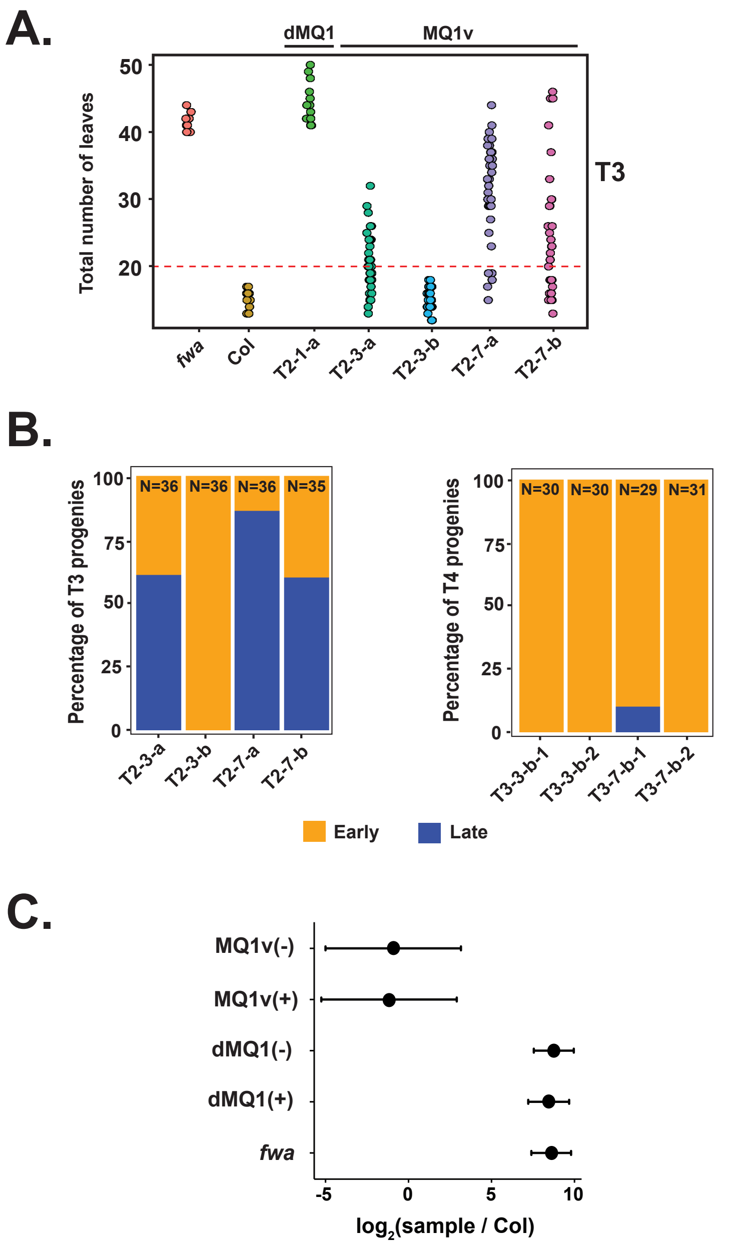

Figure S4. Heritability of targeted DNA methylation in T3 plants and *FWA* expression in T4 plants. A) Dot plot of leaf count at flowering time for T3 MQ1v and dMQ1 plants. T2 parents are labeled on the bottom of the figure. *fwa* and Col plants are included as controls**. B)** Stacked bar plot showing the percentage of T3 (left panel) and T4 (right panel) early and late flowering plants in the progeny of four different T2 and T3 early flowering lines, respectively. Percentage of early flowering plants is denoted in the bar plots. The number of progeny plants assayed (N) for each line were 36, 36, 36, 35, 30, 30, 29, and 31. **C)** *FWA* expression in *fwa*, early-flowering T4 MQ1v plants both with (+) and without (-) the MQ1v transgene, and matched dMQ1 (also +/- transgene). The x-

axis indicates log_2(genotype / Col) for each genotype indicated along the y-axis, and error bars correspond to +/- 1 standard error across 3 replicates per genotype, both calculated using DESeq2 (*27*).

Figure S5.

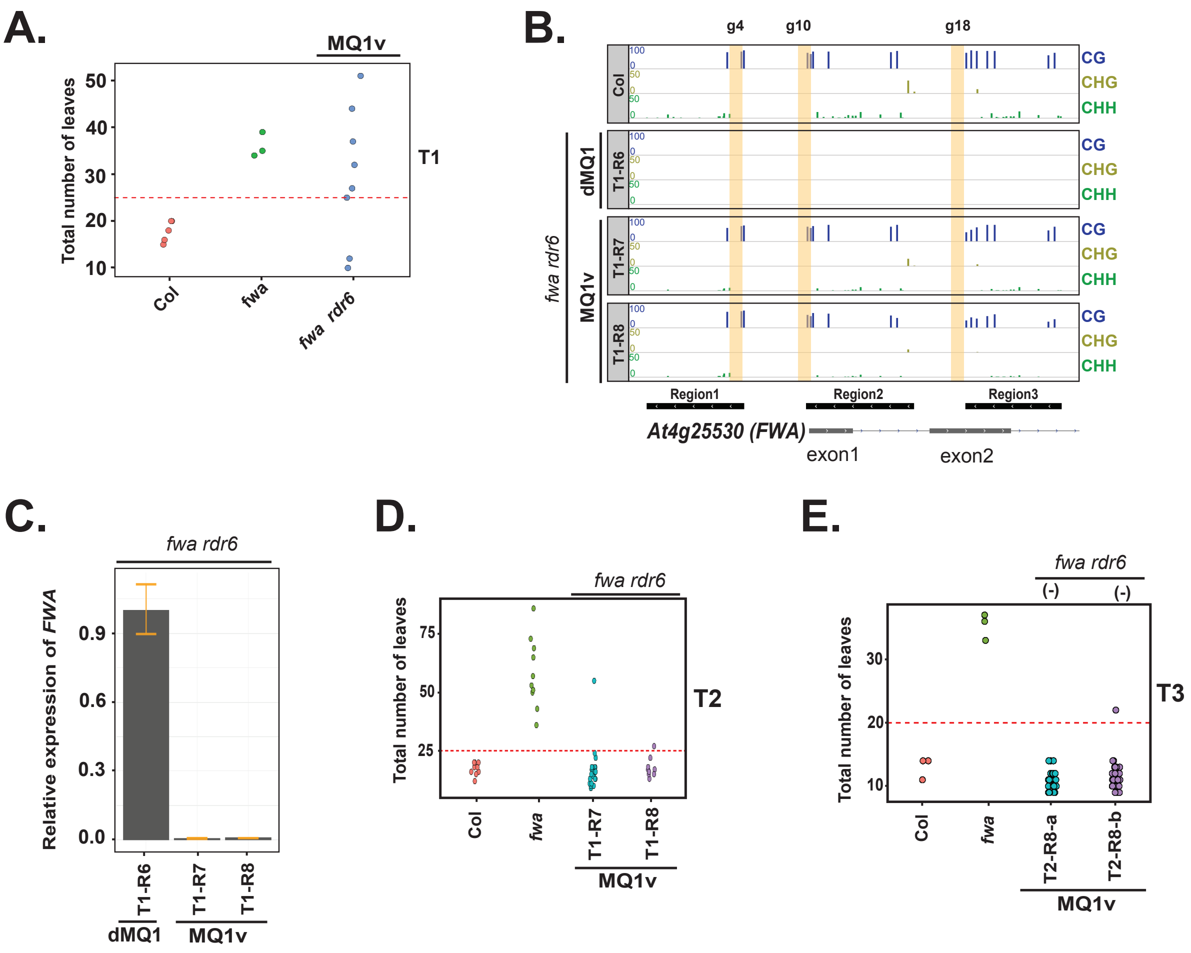

Figure S5. MQ1v targeted DNA methylation in an *rdr6* mutant background. A) Dot plot of leaf count at flowering time for T1 plants transformed with dCAS9-MQ1v in the *fwa rdr6* background. *fwa* and Col plants are included as controls**.** Each T1 plant is the result of an independent transgenic event. **B)** Bisulfite PCR sequencing of two early-flowering T1 MQ1v lines in the *fwa rdr6* background (T1-R7 and T1-R8) over three regions of the *FWA* promoter region: region 1 (Chr4:13038143 to 13038272), region 2 (Chr4:13038356 to 13038499) and region 3 (Chr4:13038568 to 13038695). A dMQ1 T1 plant was used as a negative control (T1-R6). Vertical yellow bars indicate the locations of the guide 4, guide 10, and guide 18 binding sites **C)** *FWA* qPCR expression analysis in two early-flowering T1 MQ1v plants in *fwa rdr6*, relative to a dMQ1 negative control. Error bars indicate standard deviations (n=3 technical replicates). **D-E**) Dot plot of leaf count at flowering time for T2 MQ1v plants (d) and T3 MQ1v plants (e) in *fwa rdr6* mutant backgrounds. The name of the T1 (D) and T2 (E) parent is listed at the bottom of the figure. The two T2 parent plants in (E) lacked the MQ1v construct by PCR based genotyping. *fwa* and Col plants are included as controls**.**

Figure S6.

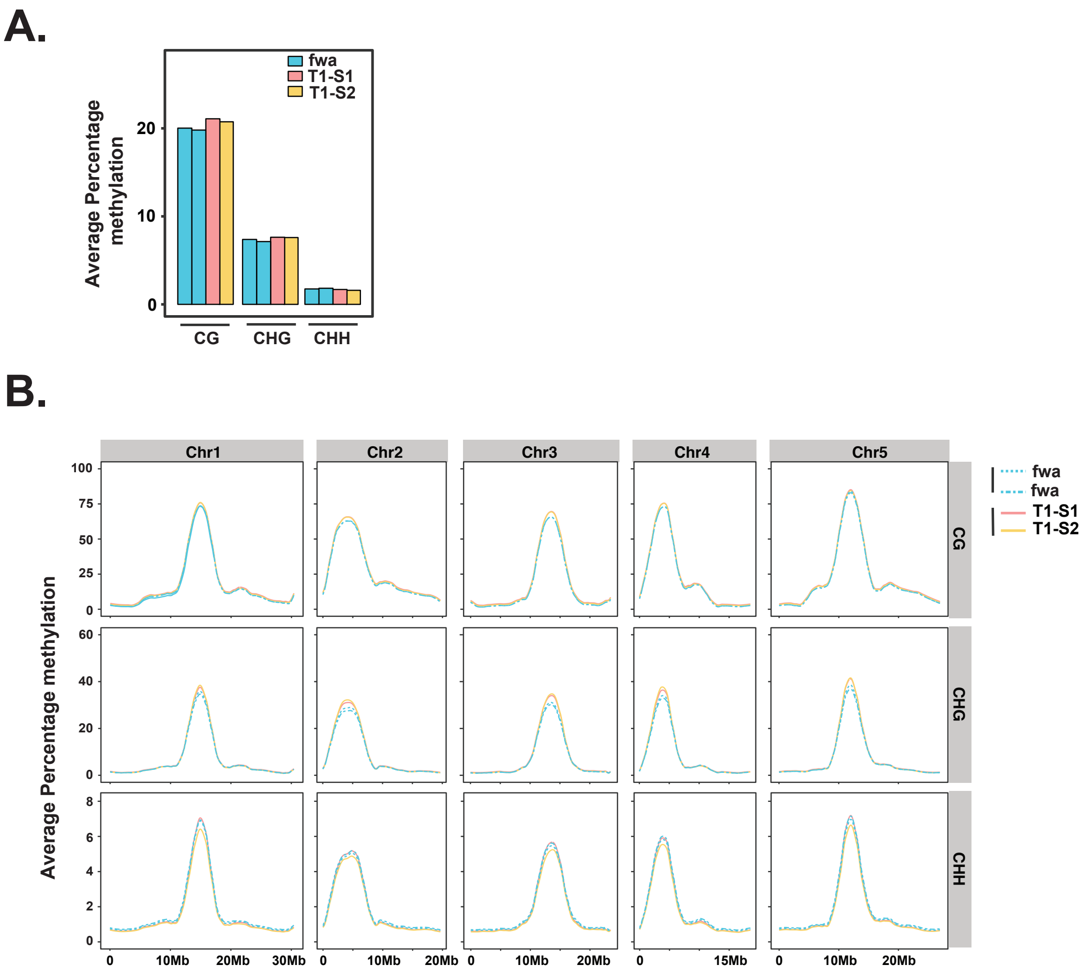

Figure S6. Genome-wide DNA methylation patterns in SunTag-MQ1v transformed T1 plants relative to a *fwa* control. Comparison of whole-genome methylation patterns in two T1 early-flowering transgenic plants (T1-S1, T1-S2) compared to a *fwa* control. **A)** Bar graphs showing whole-genome methylation percentages. **B)** Average methylation levels over each chromosome, obtained via locally weighted regression of average methylation levels over 50 kb bins (see methods).

Figure S7.

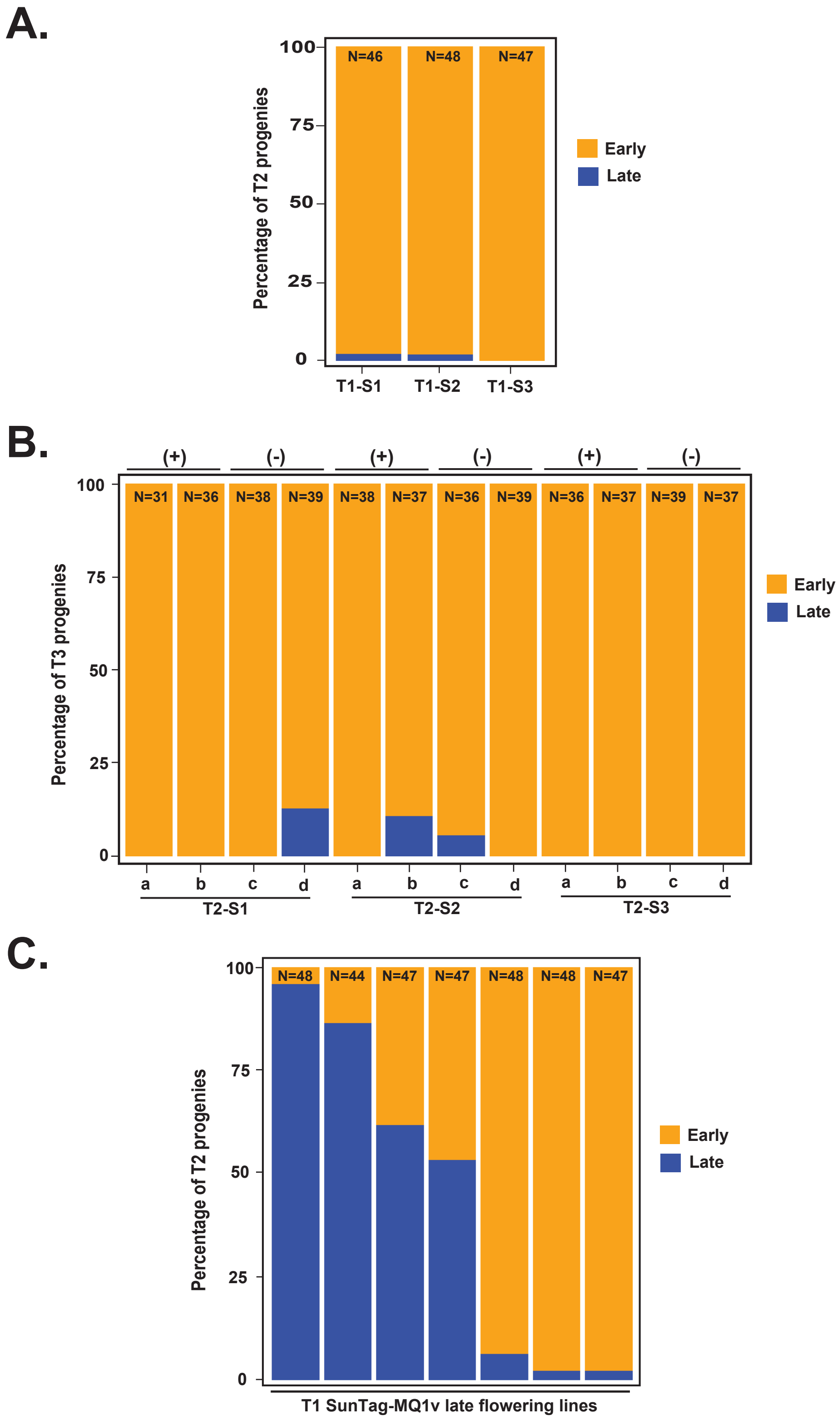
A.
Percentage of T2 progenies
100
75
50
25
0
N=46
N=48
N=47
T1-S1
T1-S2
T1-S3
Early
Late
B.
(+)
(-)
(+)
(-)
(+)
(-)
Percentage of T3 progenies
100
75
50
25
0
N=31
N=36
N=38
N=39
N=38
N=37
N=36
N=39
N=36
N=37
N=39
N=37
a
b
c
d
a
b
c
d
a
b
c
d
T2-S1
T2-S2
T2-S3
Early
Late
C.
Percentage of T2 progenies
100
75
50
25
0
N=48
N=44
N=47
N=47
N=48
N=48
N=47
T1 SunTag-MQ1v late flowering lines
Early
Late

Figure S7. Percentage of early flowering T2 and T3 SunTag-MQ1v plants. A-B) Stacked bar plot showing the percentage of early and late flowering plants among the T2 (A) and T3 (B) progeny of three T1 and twelve T2 early flowering lines. Transgene positive and negative T2 parents are denoted by symbols plus (+) and minus(-), respectively. The number (N) of T2 plants assayed for each line were 46, 48, 47. The number (N) of T3 plants assayed for each line were 31, 36, 38, 39, 38, 37, 36, 39, 36, 37, 39, and 37. **C**) Stacked bar plot showing the percentage of early and late flowering plants among the T2 progeny of seven late flowering T1 Suntag-MQ1v plants. The number (N) of progeny plants assayed for each line were 48, 44, 47, 47, 48, 48, and 47. Percentage of early flowering plants is denoted in the bar plots.

Figure S8.

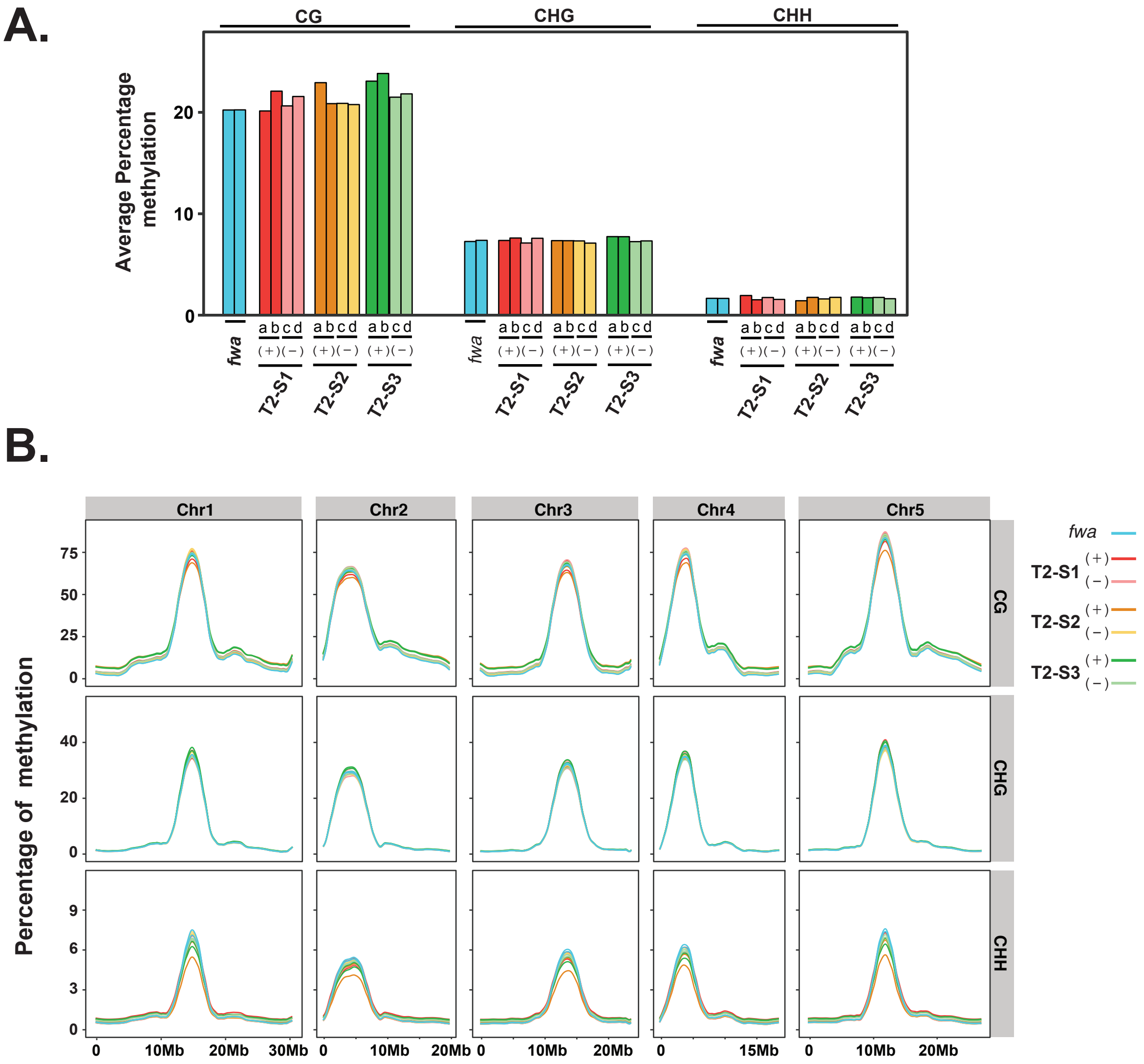

Figure S8. Genome-wide DNA methylation patterns in T2 progeny of SunTag-MQ1v transformed T1 plants. Comparison of whole-genome methylation patterns in six T2 early-flowering transgene positive (T2-S1-a, T2-S1-b, T2-S2-a, T2-S2-b, T2-S3-a, and T2-S3-b) and six transgene negative (T2-S1-c, T2-S1-d, T2-S2-c, T2-S2-d, T2-S3-c, and T2-S3-d) plants compared to a *fwa* matched control. **A)** Bar graphs showing whole-genome methylation percentages. **B)** Average methylation levels over each chromosome, obtained via locally weighted regression of average methylation levels over 50 kb bins (see methods).

Figure S9.

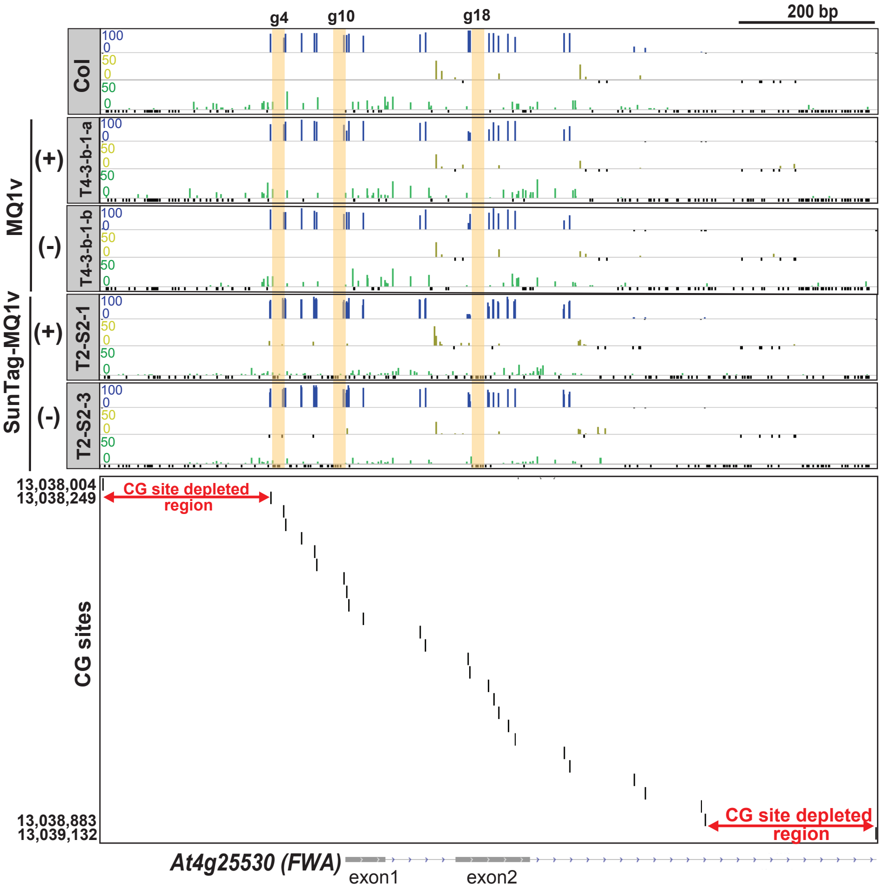

Figure S9. Comparing DNA methylation patterns introduced by MQ1v and SunTag-MQ1v. (top) DNA methylation profile at the *FWA* promoter region in two T4 MQ1v and two T2 SunTag-MQv early-flowering plants, relative to Col wild type control. Transgene positive and negative plants are denoted by symbols plus (+) and minus(-), respectively. **(bottom)** Locations of all CG sites in the region Chr4:13038004-13039132; each CG site is shown as a vertical bar. Flanking CG-depleted regions are highlighted. Vertical yellow bars indicate the locations of the guide 4, guide 10, and guide 18 binding sites.
